# Exploring the synergy of logistics, finance, and technology on innovation

**DOI:** 10.1038/s41598-024-72409-9

**Published:** 2024-09-19

**Authors:** Chunfang Wang, Md. Mominur Rahman, Abu Bakkar Siddik, Zheng Guang Wen, Farid Ahammad Sobhani

**Affiliations:** 1 College of E-Commerce and Logistics, Henan Polytechnic, Zhengdong New District, Zhengzhou, 450018 Henan Province China; 2 Bangladesh Institute of Governance and Management (BIGM), University of Dhaka (Affiliated), Dhaka, 1207 Bangladesh; 3 School of Management, University of Science and Technology of China, Jinzhai Road, Hefei, 230026 China; 4 School of Economics and Management, Shaanxi University of Science and Technology, Xi’an, 710021 Shaanxi China; 5 School of Business and Economics, United International University, Dhaka, 1212 Bangladesh

**Keywords:** Green logistics, Green finance, Green technology, Green work environment, Green innovation, Environmental sciences, Environmental social sciences, Environmental economics

## Abstract

As global environmental challenges intensify, manufacturing firms face increasing pressure to innovate sustainably. Green innovation, characterized by the development of environmentally friendly products, processes, and technologies, has become essential for firms striving to remain competitive. This study aims to investigate the influence of key factors—green logistics, green finance, and green technology—on green innovation within manufacturing firms, while exploring the mediating role of green technology in these relationships. A multi-method approach was employed, combining partial least squares structural equation modeling, fuzzy-set qualitative comparative analysis, and necessity condition analysis. 447 responses were collected from manufacturing companies in Dhaka city, Bangladesh, using structured questionnaires. The analysis revealed that green logistics and green finance have a significant positive impact on green innovation, while the influence of the green work environment was found to be positive but statistically insignificant. Additionally, green technology was identified as a significant mediator in the relationships between green finance, green logistics, and green innovation. This study offers a comprehensive green innovation model while green technology is a mediator. Furthermore, this study advances the resource-based view theory by integrating green technology as a pivotal resource that enhances a firm's competitive advantage in sustainable markets. By adopting a multi-method approach, this research provides a rigorous examination of the research questions, offering a comprehensive understanding of the dynamic interactions between green finance, green logistics, and green technology in driving innovation. Thus, this research has thought provoking implications to prioritize investments in green finance, logistics, and technology, manufacturing firms can enhance their competitiveness, improve operational efficiency, and meet evolving environmental regulations and consumer preferences.

## Introduction

In recent years, the global community has faced mounting pressure to address environmental challenges such as climate change, resource depletion, and pollution. These issues pose significant threats to ecosystems, human health, and socio-economic stability, necessitating urgent action from governments, businesses, and individuals worldwide^1–3^. Moreover, as awareness of environmental degradation grows, there has been a surge in demand for sustainable solutions across various sectors^4^. Businesses nowadays are under growing pressure to embrace eco-friendly practices and technologies, aiming to reduce their environmental footprint and support the shift towards a more sustainable economy^5^. This drive for sustainability has given rise to the idea of green innovation, which involves creating and implementing new products, methods, and business strategies that prioritize environmental protection and sustainability^6^.

The COVID-19 pandemic has highlighted the critical need for businesses to adopt green innovation, as it exposed the fragility of current economic systems and emphasized the interconnectedness of human health, environmental sustainability, and economic resilience^7,8^. The widespread disruptions caused by the pandemic forced businesses to rethink their strategies, revealing that traditional practices may not suffice in a rapidly changing world. As a result, the pandemic has acted as a catalyst, pushing firms to prioritize green innovation as a means to build more resilient and sustainable business models. This shift towards green innovation is not only crucial for environmental preservation but also enhances a company's ability to adapt and thrive in the face of future uncertainties^1,9^. In response to the challenges posed by the pandemic, manufacturing firms have adopted green innovations such as energy-efficient production processes, waste reduction technologies, and the use of renewable energy sources. These changes have not only enhanced environmental sustainability but also improved operational efficiency and resilience in the face of future disruptions.

In manufacturing companies, adopting green logistics, finance, work environment, and technology altogether offers a comprehensive way to tackle environmental issues while keeping operations efficient^10–12^. Green logistics, for instance, aims to streamline transportation, storage, and delivery methods to cut down on environmental harm, lower carbon emissions, and use resources more efficiently^13^. This may involve the use of alternative fuels, route optimization algorithms, and sustainable packaging materials to streamline supply chain operations while reducing ecological footprint. Similarly, green finance involves the allocation of capital towards environmentally sustainable projects, initiatives, and investments^14^. This may include funding for renewable energy projects, energy-efficient technologies, and eco-friendly manufacturing processes. By leveraging green finance, manufacturing companies can not only reduce their environmental footprint but also improve their financial performance and access new sources of capital^14^.

Moreover, creating a green work environment entail fostering a culture of sustainability within manufacturing companies and implementing practices that promote environmental stewardship among employees^15^. This may involve initiatives such as waste reduction programs, energy conservation measures, and eco-friendly workplace policies. By cultivating a green work environment, companies can enhance employee morale, productivity, and satisfaction while reducing their overall environmental impact^16^. Lastly, green technology involves implementing environmentally friendly technologies and innovations throughout manufacturing processes^17^. This might mean incorporating renewable energy sources, energy-efficient equipment, and eco-conscious production methods to reduce resource usage, pollution, and waste generation. Embracing green technology helps manufacturing firms improve their competitiveness, comply with regulations, and play a part in advancing towards a more sustainable future^17^.

This study is crucial to address the urgent global challenges of environmental degradation and climate change. These issues have heightened the demand for sustainable business practices, especially in the manufacturing sector^12,18–20^. As the world recognizes the necessity of transitioning to greener and more environmentally friendly operations, there's a vital need for research to uncover the factors that drive green innovation in manufacturing companies. Furthermore, the COVID-19 pandemic has highlighted the weaknesses in existing supply chains, emphasizing the importance of resilience and sustainability in business operations^8,9^. For instance, the pandemic exposed vulnerabilities such as dependency on single-source suppliers, disruptions in global logistics, and limited flexibility in adapting to sudden demand shifts. Green logistics is vital for enhancing the sustainability of supply chains by reducing carbon emissions and minimizing waste through practices like route optimization and sustainable packaging^13^. However, many firms face challenges in implementing these practices due to high initial costs and complexity in integrating new technologies^14^. Green finance, on the other hand, is crucial for funding sustainable projects, yet there are significant gaps in understanding how to effectively allocate resources and assess the financial viability of green initiatives, limiting the potential impact of these investments^21^. Against this backdrop, there is a growing imperative to develop comprehensive models that integrate green logistics, green finance, green work environment, and green technology to foster innovation and sustainability within manufacturing firms. Therefore, this study is vital for filling the gaps in existing research and offering practical insights for businesses, policymakers, and other stakeholders aiming to advance environmental sustainability and resilience in the post-pandemic period. The study aims to investigate how green logistics, finance, work environment, and technology impact green innovation within manufacturing companies. To achieve this goal, the study poses the following research questions: How does green logistics influence green innovation? What impact does green finance have on green innovation outcomes? What role does the green work environment play in driving green innovation? What is the relationship between the adoption of green technology and green innovation? and does green technology mediate the relationships between green logistics, finance, and green innovation?

This study identifies research gaps in the existing literature regarding green innovation models within manufacturing companies. Firstly, there is a notable absence of technology-driven green innovation models, with many studies focusing predominantly on organizational practices and policies without adequately considering the role of technology in driving sustainability initiatives^7,9,17^. Secondly, there is a dearth of research that identifies green technology as a mediator for green innovation outcomes. While previous studies have examined the direct effects of green technology adoption on environmental performance^18,22^, no studies explored its indirect effects on innovation outcomes within the context of green practices. Despite existing research exploring the moderating role of green technology adoption and its influence on environmental sustainability, there remains a significant gap in understanding its indirect effects on innovation outcomes within green practices^23,24^. Moreover, Hossain, et al.^25^ examined green innovation performance as an outcome, there is a need to comprehensively explore how green finance and green logistics contribute to these innovation outcomes, particularly within manufacturing firms. Thirdly, there is a lack of multimethod approaches to testing comprehensive green innovation models, with most studies employing single-method analyses such as regression or structural equation modeling^3,8,26,27^. By employing a multi-method approach integrating PLS-SEM, fsQCA, and NCA, this study aims to address these gaps and provide a more holistic understanding of the mechanisms driving green innovation within manufacturing firms. Through the integration of these methods, the study seeks to overcome the limitations of traditional single-method approaches and provide a comprehensive and nuanced analysis of the factors influencing green innovation.

## Literature review

### Green logistics and green innovation

Green logistics involves incorporating environmentally friendly practices into the logistics and supply chain management of organizations^28^. This includes strategies like optimizing transportation routes to cut down on carbon emissions, using eco-friendly packaging materials, integrating reverse logistics, assessing the carbon footprint of the supply chain, and adopting energy-efficient warehousing practices. The relationship between green logistics and green innovation is mutually beneficial because adopting sustainable logistics practices can drive innovation in environmental sustainability within manufacturing companies^13^. By integrating green logistics principles into their operations, companies not only reduce their environmental impact but also spur innovation by requiring the development of new technologies and practices to support sustainable logistics processes^10^. For example, the implementation of reverse logistics systems, which involve the return and recycling of products and materials, requires the development of innovative technologies and processes to facilitate efficient and environmentally friendly product returns and recycling.

However, although implementing green logistics practices can open doors for sustainability innovation, the actual environmental impact of these innovations may differ based on factors like organizational culture, regulations, and market demand^28^. Moreover, the effectiveness of green innovation initiatives can also be affected by external factors beyond logistics operations, including technological readiness, financial limitations, and stakeholder involvement^29^. Therefore, while green logistics can provide a conducive environment for fostering innovation in environmental sustainability, the realization of green innovation outcomes may be contingent upon a range of internal and external factors beyond the scope of logistics operations alone. Thus, the study postulated H1:

#### H1

Green logistics affect green innovation.

### Green finance and green innovation

Green finance encompasses financial mechanisms and instruments that support environmentally sustainable initiatives and projects^17,21^. This may include investments in renewable energy infrastructure, green bonds, corporate green finance policies, eco-friendly funding accessibility, environmental incentives, sustainable development loans, and other forms of financing aimed at promoting environmental sustainability^11,17,21^. The relationship between green finance and green innovation is complex, as financial support and incentives play a vital role in promoting sustainable practices within organizations^30^. Having access to green finance allows companies to invest in researching and developing environmentally friendly technologies, implementing energy-efficient processes, and adopting sustainable business practices^31^. By offering financial resources and incentives for innovation, green finance can speed up the adoption of sustainable technologies and practices, thereby encouraging green innovation^21,30^.

However, even though financial support can help drive sustainability innovation, the effectiveness of green finance initiatives in achieving green innovation outcomes can be influenced by various factors such as market conditions, regulations, and a company's capabilities^31^. Additionally, merely having access to green finance doesn't guarantee successful innovation outcomes. Implementing green innovation initiatives often requires additional factors like technological know-how, organizational dedication, and involvement from stakeholders^17^. Moreover, challenges like accessing capital, high investment expenses, and uncertainty about returns on investment can hinder the adoption of green finance and impede the achievement of green innovation goals^30^. Therefore, while green finance can stimulate green innovation, its impact depends on a variety of internal and external factors that shape the innovation process within manufacturing companies. As a result, the study proposes the following hypothesis:

#### H2

There is an association between green finance and green innovation.

### Green work environment and green innovation

The concept of a green work environment encompasses various initiatives and practices aimed at promoting sustainability and environmental responsibility within the workplace^15^. This may include measures such as energy-efficient buildings, eco-friendly workplace, green building standards implement, telecommuting promotion, employee sustainability engagement, waste reduction programs, eco-friendly policies, and employee engagement in sustainability initiatives^15,16^. The relationship between the green work environment and green innovation lies in their shared goal of fostering sustainability and environmental stewardship within organizations^16^. A conducive green work environment can serve as a catalyst for innovation by providing the necessary infrastructure, resources, and culture to support sustainable practices and encourage creative problem-solving in addressing environmental challenges^32^. Employees working in a green work environment may feel more motivated and empowered to contribute to sustainability efforts, leading to increased innovation in sustainable processes, products, and services within manufacturing companies^33^.

Creating a green work environment can foster an atmosphere conducive to innovation, but its impact on green innovation outcomes can differ based on factors like organizational culture, leadership dedication, and employee involvement^32^. Moreover, the influence of the green work environment on innovation may be affected by other contextual elements such as market conditions, technological abilities, and regulatory limitations^33^. Furthermore, the adoption of green work environment practices may not automatically translate into tangible innovation outcomes if there is a lack of alignment with broader organizational goals, limited resources, or resistance to change within the organization^15,16^. Therefore, while a green work environment can contribute to fostering a culture of sustainability and innovation, its influence on green innovation outcomes may be contingent. Therefore, H3 is suggested:

#### H3

Green work environment influences green innovation.

### Green technology and green innovation

Green technology involves implementing innovative solutions and technologies aimed at reducing environmental impact, enhancing resource efficiency, and promoting sustainability across various sectors, including manufacturing^29^. The relationship between green technology and green innovation is fundamental, as green technology acts as a primary driver of green innovation within manufacturing companies^17^. By harnessing advancements in green technology, organizations can develop and adopt innovative solutions that tackle environmental challenges while also driving business growth and competitiveness^22^. Green technology provides the tools and capabilities needed to design and manufacture sustainable products, streamline production processes, and minimize environmental impact throughout the value chain^17^. Consequently, the adoption and integration of green technology are critical in spurring green innovation by empowering companies to explore new opportunities, enhance efficiency, and distinguish themselves in the market through eco-friendly practices^29,34^.

The availability and adoption of green technology can promote innovation in sustainable practices and products, but its impact depends on various factors such as organizational culture, technological capabilities, market demand, and regulatory frameworks^34^. Additionally, successfully implementing green technology requires aligning it with organizational goals, allocating adequate resources, and implementing effective change management processes to overcome potential barriers and challenges^35^. Moreover, merely adopting green technology doesn't guarantee innovation success; it's the integration of technology with organizational processes, human capital, and strategic vision that ultimately drives meaningful innovation outcomes in sustainability within manufacturing companies^17,35^. Thus, the study proposes the following hypothesis:

#### H4

Green technology influences green innovation.

### Green logistics and green technology

Green logistics focuses on streamlining transportation, distribution, and supply chain processes to minimize environmental impact and promote resource efficiency^10^. Conversely, green technology encompasses a broad range of innovative solutions and technologies aimed at reducing environmental footprint and enhancing sustainability across various operational areas, including logistics^29^. The relationship between green logistics and green technology is symbiotic: advancements in technology often enable more efficient and environmentally friendly logistics practices, while the adoption of green logistics principles drives the need for innovative technological solutions to support sustainable operations^28,29^. For instance, integrating telematics, GPS tracking systems, and data analytics technologies into logistics operations can enable real-time monitoring of vehicle emissions, optimize routes for fuel efficiency, and reduce carbon footprint in transportation activities. Similarly, the advancement of electric vehicles, alternative fuels, and autonomous transportation technologies provides opportunities to further improve the environmental performance of logistics operations, aligning with the objectives of green logistics initiatives^10,13^.

While technological advancements hold the potential to revolutionize logistics practices and promote sustainability, the successful implementation of green technology in logistics operations may face challenges such as high initial investment costs, compatibility issues with existing infrastructure, and limited availability of specialized skills and expertise^17,22^. Additionally, the adoption of green technology in logistics may require changes in organizational processes, supply chain dynamics, and stakeholder collaboration, which could pose barriers to implementation^35^. Furthermore, the effectiveness of green logistics initiatives may also be contingent upon factors such as regulatory frameworks, market demand for sustainable products, and consumer preferences, which may influence the prioritization and adoption of green technology solutions in logistics operations^22^. Therefore, the study offers H5:

#### H5

Green logistics affects green technology.

### Green finance and green technology

Green finance plays a crucial role in helping organizations adopt and implement green technology by providing the necessary funding, resources, and incentives for sustainable initiatives^31^. Using various financial tools such as green bonds, sustainable loans, and venture capital investments, green finance allows manufacturing companies to invest in research and development, acquire state-of-the-art technologies, and integrate sustainable practices throughout their operations^30^. Additionally, green finance encourages collaboration among financial institutions, businesses, and policymakers to develop creative financing models and incentives that promote the adoption of green technologies, thus hastening the transition towards a more sustainable future^14^.

Despite their interdependence, green finance and green technology may not always align perfectly, and several factors can influence the relationship between the two^30^. One potential challenge is the availability and accessibility of green finance options, which may vary depending on market conditions, regulatory frameworks, and investor preferences^14^. While there is growing interest and demand for sustainable investments, manufacturing companies may encounter obstacles in accessing affordable green finance options, particularly for small and medium-sized enterprises (SMEs) or businesses operating in emerging markets. Additionally, mismatches between the timing and scale of financial investments and technological developments can pose challenges in effectively deploying green technologies within manufacturing operations^31^. Moreover, the complexity and novelty of green technologies may present uncertainties and risks for investors, leading to hesitancy or reluctance in allocating capital towards green technology projects^14^. Despite these potential barriers, strategic collaborations between financial institutions, technology providers, and industry stakeholders can help bridge the gap between green finance and technology, unlocking new opportunities for sustainable innovation and growth in the manufacturing sector. Thus, the study suggests H6:

#### H6

Green finance affects green technology.

### Green work environment and green technology

A conducive green work environment promotes the adoption and integration of green technologies by fostering a culture of sustainability, providing the necessary infrastructure and resources, and encouraging employee engagement and participation in eco-friendly initiatives^15^. Through initiatives such as energy-efficient buildings, waste reduction programs, and sustainable procurement practices, organizations can create an environment that supports the implementation and utilization of green technologies. Moreover, investments in employee training and education on green technology usage and best practices can enhance workforce skills and competencies, facilitating the effective deployment and management of green technology solutions. Additionally, a green work environment can serve as a catalyst for innovation by fostering collaboration, creativity, and knowledge-sharing among employees, leading to the development of new ideas and solutions for sustainable manufacturing processes and products^16^.

Retrofitting facilities and upgrading equipment to accommodate green technologies may require significant investments of time and capital, posing barriers to adoption for some manufacturing companies^35^. Moreover, resistance to change and organizational inertia may hinder the adoption and acceptance of new green technologies among employees, especially if they perceive disruptions to established workflows or job roles. Additionally, the effectiveness of green technology solutions may be contingent on factors such as workforce skill levels, management support, and organizational culture, highlighting the importance of addressing human and organizational factors alongside technological advancements^3^. Despite these challenges, strategic initiatives to promote sustainability and green practices within the workplace can create synergies between green work environment and technology, driving continuous improvement and innovation towards a more sustainable future^8^. Thus, the study postulates H7:

#### H7

Green work environment affects green technology.

### Mediating role of green technology

The mediating role of green technology in the relationship between various factors and green innovation is crucial for understanding the mechanisms through which sustainable practices drive innovation. Firstly, green logistics can influence green innovation through its impact on the adoption and integration of green technology throughout the supply chain^21^. By implementing environmentally friendly transportation methods, optimizing routing and distribution processes, and adopting eco-friendly packaging solutions, companies can reduce their carbon footprint and resource consumption, leading to more sustainable operations. Green technology, such as electric vehicles, route optimization software, and eco-friendly packaging materials, plays a vital role in enabling these green logistics practices, thereby facilitating innovation in environmentally sustainable business practices and products^19^.

Secondly, green finance can indirectly impact green innovation by facilitating the adoption and deployment of green technology solutions within manufacturing companies^20^. Access to green financing mechanisms, such as sustainability-linked loans, green bonds, and government incentives for sustainable investments, can provide the necessary capital and resources to implement green technology initiatives. These investments in renewable energy, energy-efficient equipment, and sustainable manufacturing processes contribute to improved environmental performance and innovation in green products and services. Green technology acts as a mediator in this relationship by enabling the implementation of sustainable practices funded by green finance, thus driving innovation towards greener and more sustainable outcomes^6^.

Lastly, the green work environment can influence green innovation through its interactions with green technology within manufacturing companies^3^. A supportive and conducive work environment that promotes sustainability practices and encourages employee engagement in eco-friendly initiatives can enhance the adoption and utilization of green technology solutions. By investing in employee training, providing access to resources and infrastructure, and fostering a culture of sustainability, organizations can empower their workforce to embrace green technology and drive innovation towards sustainable outcomes^31^. Green technology serves as a mediator in this relationship by facilitating the implementation and effectiveness of sustainability initiatives within the workplace, thereby contributing to innovation in green practices and products. Thus, the following hypotheses are proposed:

#### H8

Green technology mediates the link between green logistics and green innovation.

#### H9

The link between green finance and green innovation is mediated by green technology.

#### H10

The relationship between green work environment and green innovation is mediated by green technology.

### Theoretical framework

According to the Resource-Based View (RBV) theory, a company's competitive edge and success are shaped by its distinct set of strategic resources and abilities^36^. In the realm of green innovation, green technology stands out as a vital strategic resource that can be incorporated into the RBV framework^37^. Green technology encompasses a wide range of environmentally friendly technologies, processes, and practices that enable firms to reduce their environmental impact, enhance operational efficiency, and develop sustainable products and services^38^. By integrating green technology into its resource portfolio, a firm can leverage it as a source of competitive advantage by differentiating its offerings, reducing costs, and enhancing its reputation as a socially responsible and environmentally conscious organization^39^.

**Fig. 1:**
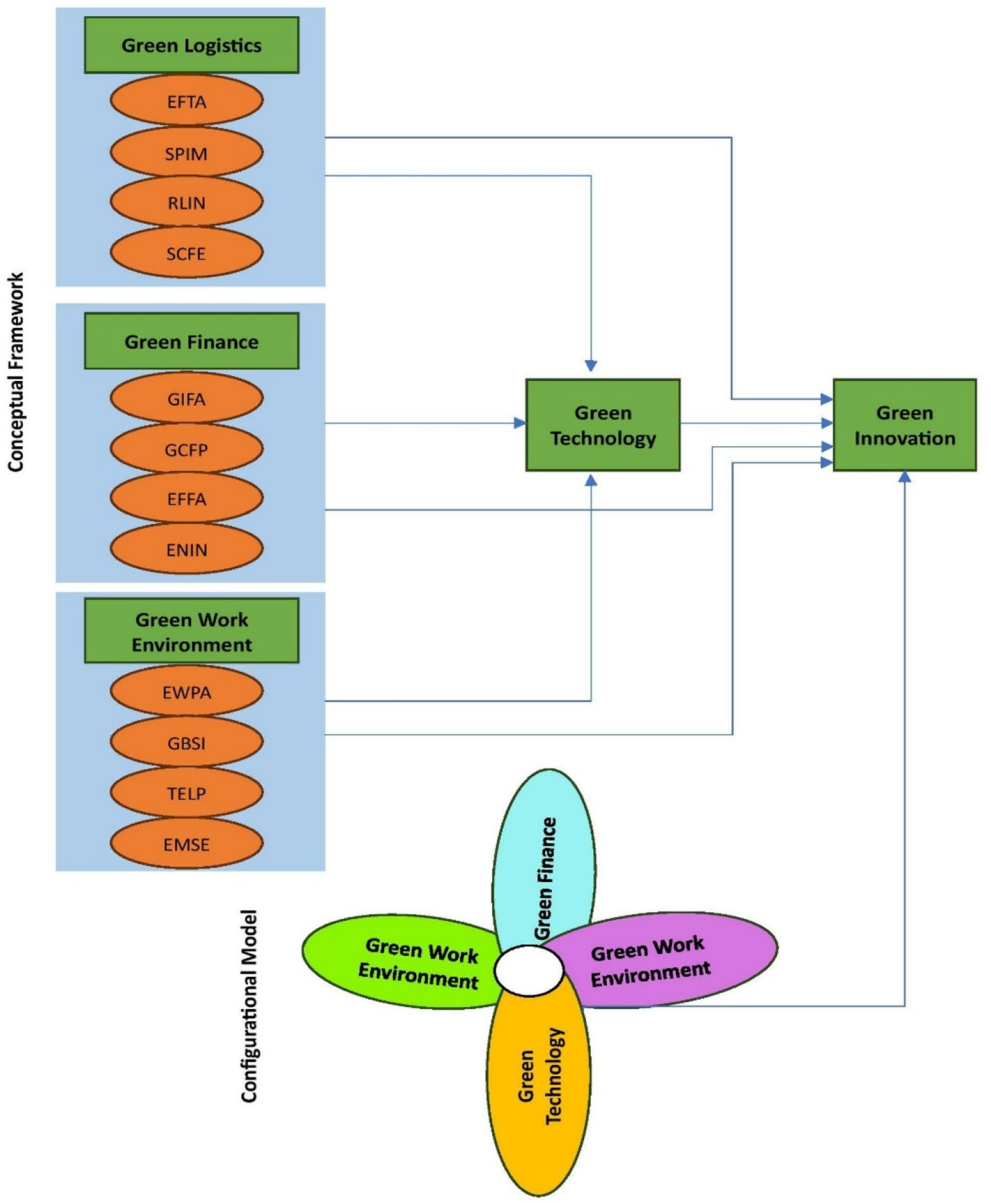
Conceptual framework and configurational model.

The association between RBV theory and fsQCA suggests a configurational approach to understanding the role of green technology in driving green innovation^37^. Figure 1 illustrates the green innovation model through a conceptual framework and configurational model of fsQCA, highlighting the interplay between different factors and their configurations in achieving high levels of green innovation. In this context, RBV theory suggests that firms must strategically manage their resources, including green technology, to achieve sustainable competitive advantage^39^. By adopting a configurational perspective, fsQCA allows for the identification of complex combinations of factors, including green technology, that are necessary for achieving desired outcomes, such as high levels of green innovation.

Strategically integrating green technology into the RBV framework and leveraging fsQCA's configurational approach can provide firms with insights into the specific combinations of factors that drive green innovation^37^. By identifying the configurations of resources, capabilities, and environmental practices that lead to superior performance in sustainable markets, firms can develop targeted strategies to enhance their competitiveness and profitability^36,38^. Moreover, by aligning their resource allocation decisions with the configurations identified through fsQCA, firms can optimize their investments in green technology and other critical resources, thereby enhancing their ability to innovate and succeed in the rapidly evolving landscape of sustainability.

Figure 1 is developed to show the conceptual and configured models. In the Fig. 1, the following abbreviations are used: EFTA: Eco-friendly Transportation Adoption, SPIM: Sustainable Packaging Implementation, RLIN: Reverse Logistics Integration, SCFE: Supply Chain Carbon Footprint Efficiency, GIFA: Green Investment Fund Availability, CGFP: Corporate Green Financing Policies, EFFA: Eco-friendly Funding Accessibility, ENIN: Environmental Incentives, EWPA: Eco-friendly Workplace Practices Adoption, GBSI: Green Building Standards Implementation, TELP: Telecommuting Promotion, and EMSE: Employee Sustainability Engagement.

## Research design

### Sampling and data collection

This study focuses on manufacturing companies located in Dhaka city, Bangladesh. Dhaka's status as a central hub for manufacturing activities in the country, with its diverse industries, makes it an ideal target for this research^26^. Additionally, Dhaka faces significant environmental challenges, which highlight the importance of assessing the adoption of green practices within the manufacturing sector^26^. By honing in on this specific population, the study aims to shed light on how green logistics, finance, work environment, and technology impact green innovation. This research not only contributes to academic understanding but also provides practical insights that can benefit Dhaka's manufacturing sector and beyond.

In this study, all methods were carried out in accordance with the relevant guidelines and regulations of Helsinki. The experimental protocols were thoroughly reviewed and approved by the Institutional Review Board of Bangladesh Institute of Governance and Management. Furthermore, informed consent was obtained from all participants involved in the study, or from their legal guardians, ensuring that their participation was voluntary and based on a full understanding of the research objectives and procedures.

The survey administration process involved distributing questionnaires via email to designated individuals, typically owners or managers, within selected manufacturing companies. A comprehensive cover letter accompanied each survey, outlining the study's objectives and inviting participation. Cement, ceramics, fuel and power, jute, pharmaceuticals and chemicals, textiles companies the number of manufacturing companies (approximately 136 in total) listed in Dhaka Stock Exchange (DSE). 25 companies were randomly chosen to receive survey invitations, totaling 500 potential respondents (20 questionnaires per company). The study used individual respondent as the unit of analysis from these manufacturing firms employing convenient sampling. Impressively, the response rate reached 91%, with 455 completed surveys returned. Following careful screening to exclude unusable or ineligible responses, a robust dataset of 447 responses remained for subsequent analysis. The demographic characteristics of the respondents are detailed in Table 1.

**Table 1:** Socio-demographic profile of respondents.

Category	Characteristics	Frequency (N = 447)	%
Gender	Male	259	58
	Female	188	42
Age	18–25	107	24
	26–35	131	29
	36–45	114	26
	46–55	66	15
	Above 55	29	6
Employment	Owner/MD/president	20	4
	CEO	22	5
	Managers	146	33
	Officers	259	58
Education	Diploma	73	16
	Bachelor	208	47
	Master	127	28
	Others	39	9
Type of manufacturer	Cements	63	14
	Ceramics	81	18
	Fuel and power	75	17
	Jute	66	15
	Pharmaceutical and chemicals	95	21
	Textiles	67	15

Table 1 presents the demographic profile of the respondents involved in the study, outlining various characteristics such as gender, age, employment position, and education level. This breakdown is essential for understanding the composition of the sample and provides insights into the diversity and representativeness of the dataset. The gender distribution indicates that 58% of the respondents are male, while 42% are female, reflecting a relatively balanced representation across genders within the sample. Age distribution highlights a broad range of respondents, with the majority falling within the age brackets of 26–35 (29%) and 36–45 (26%). However, there is also notable participation from younger age groups (18–25) and older respondents (above 55), demonstrating a diverse age profile within the sample. In terms of employment positions, most respondents are officers (58%), followed by managers (33%), and a smaller proportion comprising owners, CEOs, or presidents (9% combined). This distribution suggests that the survey captured perspectives from various organizational levels, including top management and operational roles. The education level of respondents varies, with a significant portion holding bachelor's degrees (47%) and master's degrees (28%). Additionally, there are respondents with diplomas (16%) and other educational qualifications (9%), indicating a mix of educational backgrounds within the sample.

To ensure the accuracy of our sample, we carefully analyzed potential sources of bias. Initially, we compared respondents who replied early with those who responded later using a t-test to check for non-response bias. Surprisingly, we found no significant differences in means between these groups, indicating that the timing of responses didn't skew our sample representation. Additionally, we conducted a thorough examination of common method bias (CMB) using a comprehensive collinearity test^40^. The results showed that the variance inflation factor for all constructs consistently remained below the critical threshold of 3, ranging from 1.112 to 2.103^41^. This highlights the absence of any problematic CMB effects, confirming the reliability and validity of our study's results. By addressing both non-response bias and common method bias diligently, we strengthen confidence in the trustworthiness and accuracy of our research findings.

### Measures

All survey instruments were crafted using established scales from existing literature, with slight adjustments in wording to fit the specific context of our study (refer to Table A1 in the appendix). This research employs three higher-order or second-order constructs, along with fourteen first-order or lower-order constructs. Green logistics^10,13,28^, for instance, is operationalized as a higher-order construct comprising four lower-order constructs: eco-friendly transportation adoption (EFTA), sustainable packaging implementation (SPIM), reverse logistics integration (RLIN), and supply chain carbon footprint efficiency (SCFE). Similarly, green finance^21,30,31^ is represented as a higher-order construct consisting of four lower-order constructs: green investment fund availability (GIFA), corporate green financing policies (CGFP), eco-friendly funding accessibility (EFFA), and environmental incentives (ENIN). Lastly, green work environment^15,16^ is conceptualized as a higher-order construct composed of four lower-order constructs: eco-friendly workplace practices adoption (EWPA), green building standards implementation (GBSI), telecommuting promotion (TELP), and employee sustainability engagement (EMSE). Green technology^29,34,35^ and green innovation^6,33,34,39^ are considered as the two lower-order or first-order constructs. Detailed information on constructs, items, and their sources can be found in Table A1.

### Analysis strategy

To comprehensively examine the dynamics of green innovation within manufacturing companies, a multi-method approach was employed, incorporating PLS-SEM, fsQCA, and Necessity Condition Analysis (NCA). Initially, we utilized PLS-SEM through SmartPLS 4 to examine the overall effects and mediating impacts of green logistics, green finance, green work environment, and green technology on green innovation^41^. PLS-SEM offers several advantages compared to covariance-based SEM, especially in modeling reflective-formative hierarchical latent variables and identifying significant drivers or barriers of the outcome variable^42^. The bootstrap technique integrated within PLS-SEM enhances the analysis of mediating effects, outperforming traditional methods like the causal-step approach and Sobel test^43^. In addition, we conducted fsQCA to uncover combinations of factors, known as causal recipes, that contribute to high levels of green innovation^44^. fsQCA utilizes Boolean logic to identify multiple pathways leading to the desired outcome, offering a nuanced understanding beyond the limitations of conventional symmetric approaches like regression and SEM^44^. Given the inherent complexity of firms' decision-making processes, fsQCA provides a robust framework to analyze the synergistic effects of various factors on green innovation, enhancing insights gained from net effect analysis.

Finally, we opted to employ NCA as a complementary method in our research for several reasons^45^. Firstly, NCA offers a unique perspective by focusing on identifying the essential conditions required for the occurrence of a specific outcome. This approach aligns well with our aim of understanding the critical factors driving green innovation within manufacturing firms. Secondly, integrating NCA allows us to complement our primary analytical methods, such as regression-based analyses or configurational analyses, providing a more comprehensive understanding of the determinants of green innovation^12,46^. By combining NCA with these existing techniques, we can delve deeper into the underlying mechanisms and necessary conditions that contribute to or inhibit the desired outcome. Lastly, NCA enhances the robustness of our findings by offering a complementary lens through which to examine the research problem, thereby strengthening the validity and reliability of our conclusions.

## Empirical analysis

### Analysis of PLS-SEM

#### Reflective measurement model assessment

We thoroughly examined the psychometric properties of the fourteen reflective constructs by assessing internal consistency reliability, convergent validity, and discriminant validity. To gauge internal consistency reliability, we used Dijkstra-Henseler's rho as a substitute for the traditional Cronbach's alpha (CA), providing a more lenient measure of composite reliability. The results, presented in Table 2, indicated that all CA values exceeded the recommended threshold of 0.7, indicating robust construct reliability^41^. Additionally, convergent validity was confirmed through indicator loadings and average variance extracted (AVE) values, which surpassed 0.533 for all reflective constructs. This implies that each construct's indicators reliably converge to measure the underlying construct, thereby bolstering the validity of the measurement model.

**Table 2:** Measurement model summary of reflective (lower-order) constructs.

Constructs	Items	OL	CA	CR	AVE	Constructs	Items	OL	CA	CR	AVE
Eco-friendly transportation adoption	EFTA1	0.801	0.724	0.844	0.644	Reverse logistics integration	RLIN1	0.777	0.796	0.753	0.533
	EFTA2	0.839					RLIN2	0.809			
	EFTA3	0.766					RLIN3	0.701			
Sustainable packaging implementation	SPIM1	0.796	0.716	0.841	0.638	Supply chain carbon footprint efficiency	SCFE1	0.924	0.900	0.936	0.830
	SPIM2	0.840					SCFE2	0.925			
	SPIM3	0.758					SCFE3	0.884			
Green investment fund availability	GIFA1	0.809	0.754	0.858	0.668	Eco-friendly funding accessibility	EFFA1	0.725	0.835	0.823	0.599
	GIFA2	0.808					EFFA2	0.700			
	GIFA3	0.834					EFFA3	0.819			
Corporate green financing policies	GCFP1	0.764	0.807	0.887	0.725	Environmental incentives	ENIN1	0.860	0.821	0.893	0.737
	GCFP2	0.884					ENIN2	0.866			
	GCFP3	0.899					ENIN3	0.848			
Eco-friendly workplace practices adoption	EWPA1	0.815	0.755	0.860	0.672	Telecommuting promotion	TELP1	0.744	0.819	0.842	0.739
	EWPA2	0.849					TELP2	0.864			
	EWPA3	0.794					TELP3	0.728			
Green building standards implementation	GBSI1	0.855	0.888	0.829	0.683	Employee sustainability engagement	EMSE1	0.672	0.810	0.791	0.560
	GBSI2	0.844					EMSE2	0.736			
	GBSI3	0.775					EMSE3	0.830			
Green technology	GRT1	0.849	0.838	0.826	0.664	Green innovation	GRIN1	0.728	0.838	0.822	0.695
	GRT2	0.867					GRIN2	0.717			
	GRT3	0.752					GRIN3	0.887			
	GRT4	0.717					GRIN4	0.883			

#### Formative measurement model assessment

To estimate the reflective-formative higher-order constructs (green finance, green logistics, and green work environment), we employed a two-stage approach. In the initial stage, we obtained latent variable scores for the lower-order constructs using the repeated indicator approach. These scores were subsequently used as formative indicators for the higher-order constructs in the second stage. To validate the higher-order constructs, we assessed the collinearity between formative indicators.

The data in Table 3 indicate that all formative indicators had variance inflation factors (VIF) below the critical threshold of 3, suggesting no issues with multicollinearity^41^. This implies that the formative indicators collectively contribute to the higher-order constructs without redundancy or undue influence from collinearity. Moreover, we conducted a bootstrapping analysis on the model with 5000 subsamples to evaluate the significance and relevance of the indicator weights. Table 3 shows that all indicator weights were statistically significant at the p < 0.01 level, confirming their meaningful contribution to the respective constructs. Additionally, we assessed the reliability and validity of the higher-order constructs using CA, CR, and AVE. These metrics ensure the robustness and validity of the constructs, as they met the thresholds for CA, CR, and AVE, providing further evidence of their reliability and validity.

**Table 3:** Measurement model summary of formative (higher-order) constructs.

Higher-order constructs	Formative Indicators	Weights	VIF	CA	CR	AVE
Green finance	Green investment fund availability	0.411	1.653	0.780	0.826	0.657
	Corporate green financing policies	0.392	1.589			
	Supply chain carbon footprint efficiency	0.326	1.390			
	Eco-friendly funding accessibility	0.441	1.901			
Green logistics	Eco-friendly transportation adoption	0.332	1.668	0.795	0.808	0.602
	Sustainable packaging implementation	0.347	1.440			
	Reverse logistics integration	0.301	1.066			
	Reverse logistics integration	0.426	1.855			
Green work environment	Eco-friendly workplace practices adoption	0.308	2.619	0.789	0.837	0.639
	Green building standards implementation	0.413	1.692			
	Telecommuting promotion	0.389	1.534			
	Employee sustainability engagement	0.314	2.413			

To rigorously assess discriminant validity, two widely accepted methods were employed: the heterotrait–monotrait ratio of correlations (HTMT) and the Fornell–Larcker criterion. Firstly, HTMT values were computed to gauge the distinctiveness of constructs from each other. As indicated in Table 4, all HTMT values fell below the threshold of 0.90^43^, signifying that correlations between constructs' indicators are lower than their correlations with indicators of other constructs, thus affirming discriminant validity. Secondly, the Fornell-Larcker criterion was utilized to validate discriminant validity. This criterion compares the square root of each construct's AVE with the correlations between that construct and other constructs^42^. In Table 4, it is evident that the square root of each construct's AVE (displayed along the diagonal) exceeds the correlations between that construct and other constructs (shown in the respective columns). This consistent pattern indicates that each construct shares more variance with its indicators than with indicators of other constructs, providing further evidence of good discriminant validity.

**Table 4:** Discriminant validity.

	Green finance	Green innovation	Green logistics	Green technology	Green work environment
Fornell–Larcker criterion					
Green finance	0.810				
Green innovation	0.799	0.833			
Green logistics	0.676	0.752	0.775		
Green technology	0.767	0.764	0.715	0.814	
Green work environment	0.295	0.067	0.070	0.005	0.799
HTMT ratio					
Green finance	–				
Green innovation	0.307				
Green logistics	0.688	0.275			
Green technology	0.811	0.698	0.251		
Green work environment	0.851	0.794	0.713	0.628	–

#### Structural model assessment

**Fig. 2:**
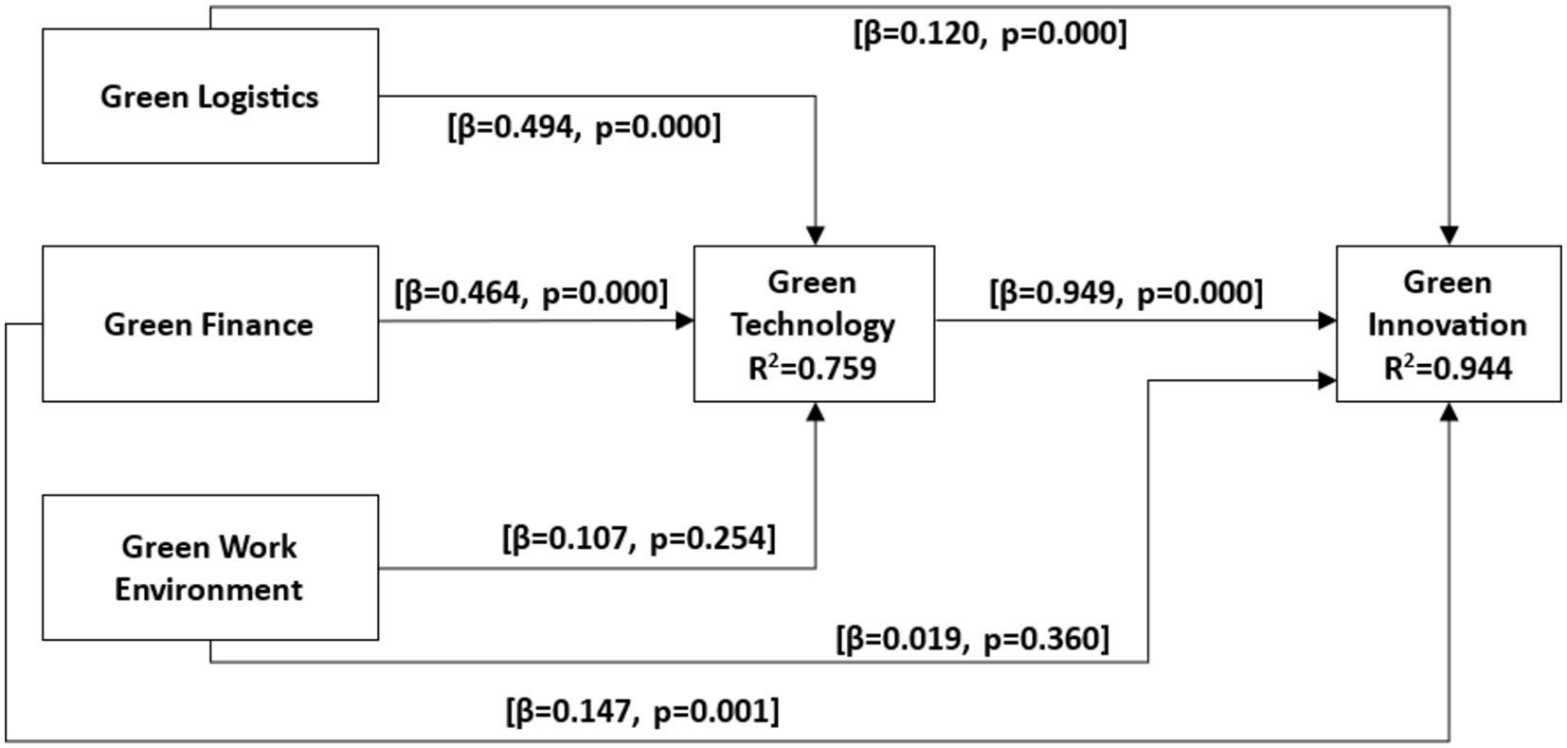
Results of structural model.

To assess the significance of the hypothesized relationships, we employed a robust non-parametric bootstrap procedure with 5000 subsamples and a confidence interval of 95 percent^41,42^. The results, summarized in Fig. 2 and detailed in Table 5, offered valuable insights into the connections between different constructs. The values for RMSEA, NFI, and SRMR were 0.06, 0.83, and 0.07, respectively. The RMSEA, NFI, and SRMR values indicates the best of the model^41,42^. Initially, the analysis revealed that green logistics had a statistically significant positive effect on green innovation (β = 0.120, p < 0.01), thus confirming the validity of H1. Similarly, green finance showed a significant positive impact on green innovation (β = 0.147, p < 0.01), providing empirical support for H2. However, the effect of the green work environment on green innovation was positive but statistically insignificant (β = − 0.019, p > 0.05), leading to the rejection of H3. The non-significant impact of the green work environment may be due to the overarching influence of work climate and culture, which are pivotal in shaping employee behaviors and innovation outcomes, potentially overshadowing direct environmental initiatives.

**Table 5:** Testing direct effects of green logistics, green finance, green work environment, and green technology on green innovations.

Relationships	Coefficients	T-statistics	Confidence interval [2.5%, 97.5%]	P values	Remarks
H1: green logistics → green innovation	0.120	4.475	[0.067, 0.161]	0.000	Yes
H2: green finance → green innovation	0.147	3.265	[0.083, 0.247]	0.001	Yes
H3: green work environment → green innovation	0.019	0.916	[− 0.061, 0.023]	0.360	No
H4: green technology → green innovation	0.949	32.453	[0.879, 0.994]	0.000	Yes
H5: green logistics → green technology	0.494	8.703	[0.400, 0.617]	0.000	Yes
H6: green finance → green technology	0.464	8.157	[0.367, 0.554]	0.000	Yes
H7: green work environment → green technology	0.107	1.140	[− 0.158, 0.216]	0.254	No

Additionally, the study revealed a significant positive impact of green technology on green innovation (β = 0.949, p < 0.01), providing robust support for H4. Furthermore, it was observed that both green logistics (β = 0.494, p < 0.01) and green finance (β = 0.464, p < 0.01) significantly influenced green technology, supporting H5 and H6, respectively. However, the analysis found that the green work environment had no significant impact on green technology (β = 0.107, p > 0.05), leading to the rejection of H7.

In the final phase of analysis, we explored the mediating role of green technology in the relationships between green logistics, green finance, and green innovation. The results illuminated the indirect effects of these constructs on green innovation through the mediating influence of green technology. Initially, the analysis unveiled a significant indirect effect of green logistics on green innovation through green technology (β = 0.469, p < 0.01). The bootstrap confidence interval, spanning from 0.381 to 0.575, further validated the robustness of this indirect effect, providing strong support for H8. Similarly, a significant indirect effect of green finance on green innovation through green technology was observed (β = 0.441, p < 0.01). This finding not only corroborated the importance of green technology as a mediator but also lent credence to H9. The confidence interval further bolstered the significance of this indirect effect. Conversely, the analysis revealed that the indirect effect of the green work environment on green innovation through green technology did not attain significance (β = − 0.102, p > 0.05), thereby failing to provide support for H10. This suggests that while green technology plays a mediating role in the relationships between green logistics, green finance, and green innovation, the green work environment does not exert a significant indirect effect on innovation outcomes through technology (Table 6).

**Table 6:** Mediating effects of green technology.

Relationships	Coefficients	T-statistics	Confidence interval [2.5%, 97.5%]	P values	Remarks
H8: green logistics → green technology → green innovation	0.469	9.384	[0.381, 0.575]	0.000	Yes
H9: green finance → green technology → green innovation	0.441	7.679	[0.338, 0.531]	0.000	Yes
H10: green work environment → green technology → green innovation	0.102	1.158	[0.151, 0.199]	0.247	No

### Analysis of fsQCA

We employed fsQCA to investigate how various factors interact to influence green innovation outcomes^47^. Following the instructions outlined in the fsQCA user's guide, we completed three essential steps: calibrating the data, constructing the truth table, and analyzing causal conditions.

In the first phase, standard data underwent a conversion into fuzzy sets (refer to Table 7) by assigning fuzzy scores to align with the criteria for full membership (fuzzy score = 0.95), cross-over anchors (fuzzy score = 0.50), and full non-membership (fuzzy score = 0.05), as recommended by Sukhov et al.^44^ and Rahman^12^. This conversion allowed us to grasp the intricate connections between variables and their impact on green innovation.

**Table 7:** Calibration.

Variable	Fully-in	Cross-over	Fully-out	Mean	Standard deviation	Minimum	Maximum	Cases
Green finance	2.130	0.000	− 2.970	0.529	0.239	0.05	0.95	447
Green logistics	2.470	0.000	− 3.380	0.521	0.216	0.05	0.95	447
Green technology	1.890	0.000	− 3.450	0.549	0.237	0.05	0.95	447
Green work environment	2.240	0.000	− 2.580	0.513	0.246	0.05	0.95	447
Green innovation	1.850	0.000	− 3.290	0.547	0.241	0.05	0.95	447

Next, our attention turned to constructing the truth table to create diverse combinations of causal conditions that are adequate for achieving significant levels of green innovation^48^. This entailed establishing a consistent cutoff value of 1 and setting the number-of-cases threshold at 3 to ensure the reliability of our analysis^45^. Subsequently, we utilized standard analytical methods to derive the "intermediate solution," incorporating partial logical remainders into the solution. This approach enabled us to identify the causal patterns leading to substantial levels of green innovation, offering valuable insights into the combined effects of multiple factors on innovation outcomes^48^. For further information and details regarding the truth table minimization process, refer to Table A2 in the Appendix, which outlines the minimized truth table resulting from our analysis.

**Fig. 3:**
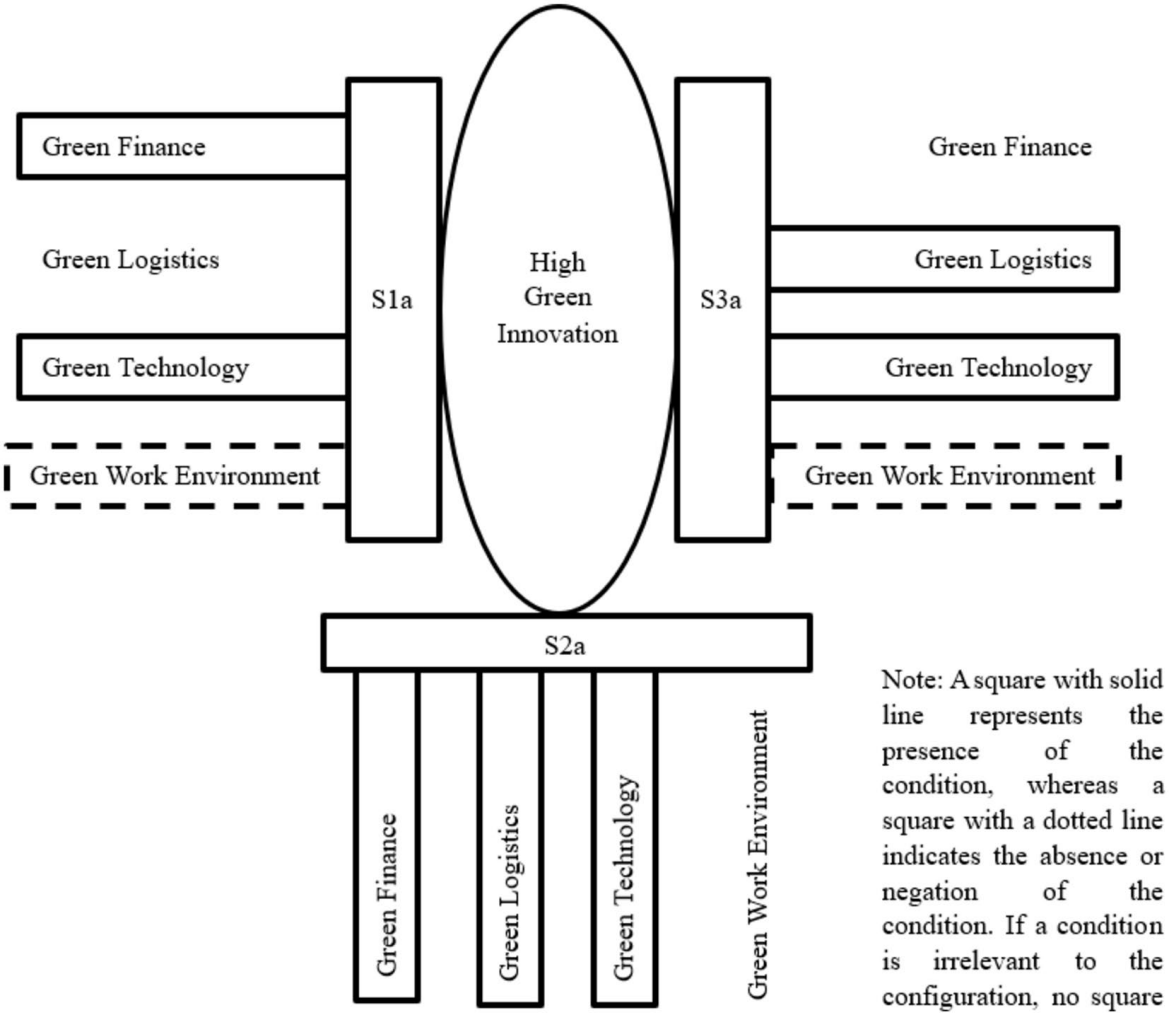
Graphical presentation of the causal configurations for high green innovation.

Table 8 and Fig. 3 present a summary of the intermediate solutions for achieving high and low levels of green innovation. In Model 1, three distinct causal configurations leading to high green innovation are identified. Each configuration demonstrates consistent values above 0.80, indicating strong causal relationships, with an overall solution coverage of 0.986, suggesting high informativeness of the model^48^. In Solution S1a, it is revealed that high levels of green innovation can be achieved despite a low green work environment, provided there are high levels of both green finance and green logistics. This underscores the importance of financial and logistical support in driving innovation, particularly in environments where green workplace practices may be lacking. Solution S2a highlights the significance of green logistics and green technology, alongside a low green work environment, in fostering high levels of green innovation. This suggests that even in the absence of robust green finance initiatives, the combination of efficient logistical processes and advanced green technologies can propel innovation in environmentally sustainable practices. Solution S3a emphasizes the pivotal role of green finance and green logistics, coupled with high levels of green technology, in achieving high green innovation outcomes. This configuration underscores the synergistic effects of financial investment and logistical support in conjunction with advanced technological solutions in driving innovation towards sustainability goals. Thus, the findings underscore the critical importance of green technology as a core condition for achieving high levels of green innovation because it is visible to all the three solutions. In Table 8, ~ indicates absent of conditions.

**Table 8:** Sufficient conditions for high (low) green innovation.

Configurations	Raw coverage	Unique coverage	Consistency
Model 1 (high): green innovation = f (green finance, green logistics, green technology, green work environment)			
Solution S1a: f (green finance*green technology* ~ green work environment)	0.877	0.018	0.907
Solution S2a: f (green logistics*green technology* ~ green work environment)	0.863	0.002	0.906
Solution S3a: f (green finance*green logistics*green technology)	0.958	0.042	0.955
Solution coverage	0.986		
Solution consistency	0.862		
Model 2 (low): ~ green innovation = f (green finance, green logistics, green technology, green work environment)			
Solution S1b: f (~ green technology)	0.945	0.026	0.949
Solution S2b: f (~ green logistics)	0.892	0.008	0.843
Solution S3b: f (~ green finance)	0.892	0.019	0.856
Solution coverage	0.987		
Solution consistency	0.794

Furthermore, Table 8 also shows that the absence of conditions can lead to low green innovation. As shown in Model 2, the absence of green technology (S1b), green logistics (S2b), and green finance (S3b) lead to low green innovation. Through the fsQCA results, it is found that green finance, green logistics, and green technology are the sufficient configurations for high green innovation regardless of green work environment. These findings are consistent with the results of PLS-SEM.

**Fig. 4:**
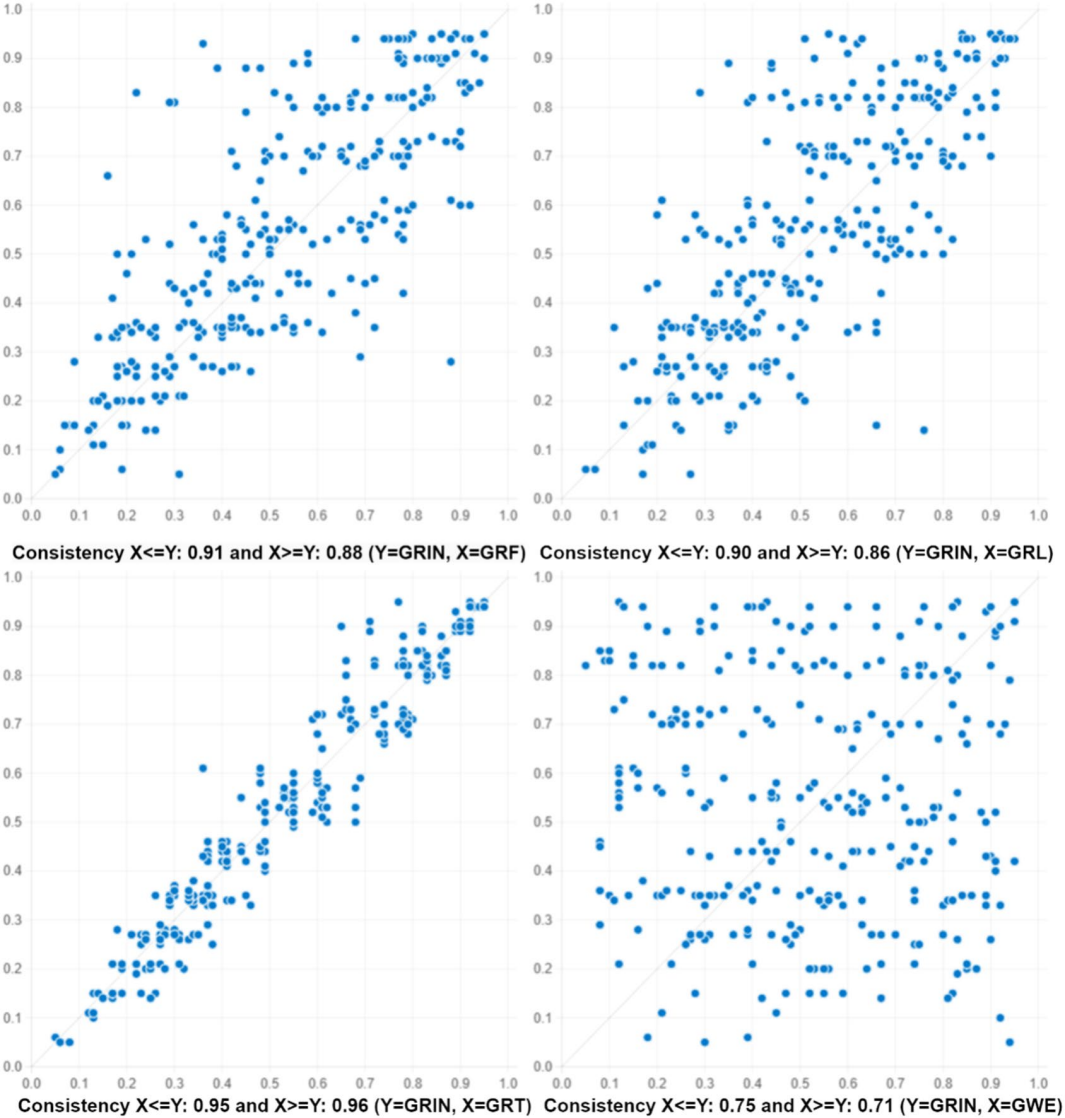
Scatter plots.

Further, Fig. 4 displays scatter plots depicting the relationships between different pairs of variables in terms of their consistency levels^46^. Each plot compares the consistency of two variables, where one variable is represented on the X-axis and the other on the Y-axis. The consistency values are shown for scenarios where the X variable is less than or equal to the Y variable (X <  = Y) and where the X variable is greater than or equal to the Y variable (X >  = Y). For instance, the scatter plot for green finance (GRF) and green innovation (GRIN) reveals a consistency of 0.91 when GRF is less than or equal to GRIN and 0.88 when GRF is greater than or equal to GRIN. Similarly, other scatter plots illustrate the consistency levels between green logistics (GRL), green technology (GRT), green work environment (GWE), and green innovation (GRIN), providing insights into the relationships and interactions among these variables in driving green innovation within the context of the study.

### Analysis of NCA

NCA has become a well-established approach and data analysis technique utilized across various fields including social, medical, and technical sciences^45^. It serves to identify necessary conditions within datasets, offering valuable insights that complement regression-based methods or configurational methods like fsQCA. NCA is versatile and can be employed as a stand-alone method or integrated with other analytical approaches^44^. To perform complete and rigor analysis, First, we employ NCA of fsQCA. Second, we perform all steps of NCA stand-alone approach. A juxtaposition of the outcomes from NCA and fsQCA reveals that NCA has the capability to pinpoint a greater number of necessary conditions compared to fsQCA. Moreover, NCA provides the advantage of specifying the requisite level of each condition necessary to achieve a desired level of the outcome^45^. This highlights the nuanced insights that NCA offers in understanding the underlying factors influencing outcomes, particularly in complex contexts such as those encountered in research on green innovation within manufacturing firms.

Table 9 presents the outcomes of NCA conducted within the framework of fsQCA. In line with the criteria established by Dul^45^ and Ragin^48^, a condition is deemed necessary when its consistency value exceeds 0.9. As depicted in Table 9, the results indicate that among the studied factors—green finance, green logistics, green technology, and green work environment—only green technology emerges as essential for achieving the desired response, namely green innovation. In Table 9, ~ indicates absent of condition. Bolded values indicate necessary conditions.

**Table 9:** Necessary condition analysis for high (low) green innovation.

Conditions	High green innovation		Low green innovation	
	Consistency	Coverage	Consistency	Coverage
Green finance	0.877	0.907	0.649	0.554
~ Green finance	0.569	0.663	0.892	0.856
Green logistics	0.863	0.906	0.665	0.576
~ Green logistics	0.596	0.683	0.892	0.843
Green technology	**0.958**	**0.955**	0.649	0.533
~ Green technology	0.532	0.648	**0.945**	**0.949**
Green work environment	0.705	0.752	0.762	0.671
~ Green work environment	0.692	0.779	0.719	0.668

Significant values are given in bold.

**Fig. 5:**
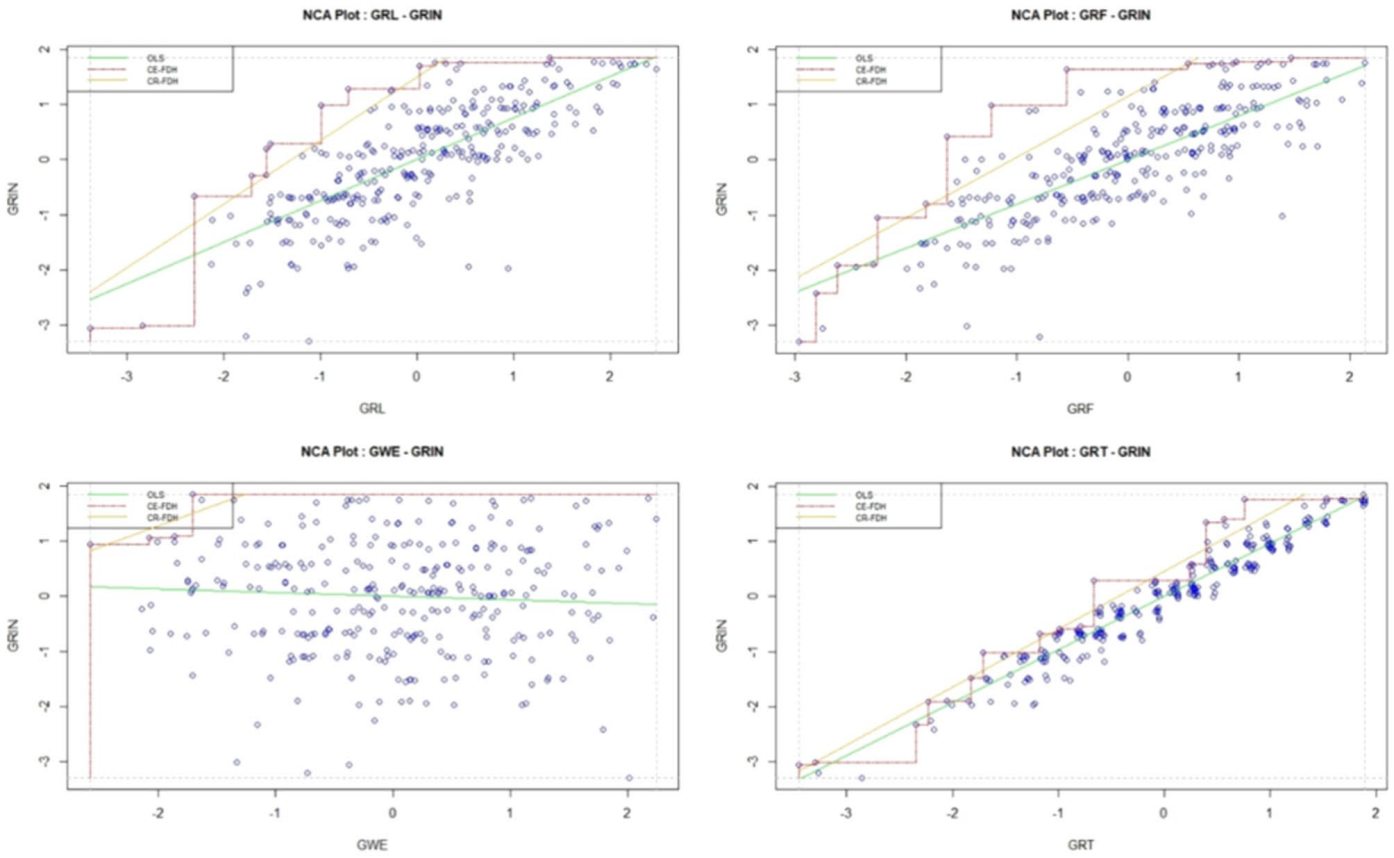
Scatter plots for X = GRL, GRF, GWE, GRT, and Y = GRIN.

Now, we proceed with the NCA process step by step, which comprises three main steps. Step 1 involves creating scatter plots. Step 2 assesses effect sizes, and Step 3 entails bottleneck table analysis. In the initial step illustrated in Fig. 5, XY scatter plots are generated, with green logistics, green finance, green technology, and green work environment plotted on the horizontal X-axis, while green innovation is plotted on the vertical Y-axis. Values increase to the right and upwards on the plot^45^. This method entails examining the amount of empty space in the upper-left corner when plotting X and Y against each other. A significant empty space in this corner suggests that achieving a high value on Y requires a minimum level of X. To determine this threshold, either a step function (CE-FDH) or a linear regression function (CR-FDH) is applied to the ceiling points with a higher y-value than all points with a lower x-value, as indicated by the dots in Fig. 5. This approach enables the identification of critical thresholds and relationships between variables, facilitating a deeper understanding of the factors influencing green innovation.

In the second step, we evaluate the significance of the effect size (d) within our specific context to determine its theoretical or practical relevance. Following general benchmarks, where 0 < d < 0.1 indicates a "small effect," 0.1 ≤ d < 0.3 signifies a "medium effect," 0.3 ≤ d < 0.5 represents a "large effect," and d ≥ 0.5 indicates a "very large effect," we assess the magnitude of the observed effects^12,45^. Additionally, we compare the accuracy of the effect size against the benchmark of 95%. In our study, we consider the necessity effect size as meaningful, comprising a combination of large, medium, and small effect sizes. However, it's important to acknowledge that the accuracy might be somewhat compromised due to the limited number of observations, with certain data points exceeding the ceiling line. Despite these constraints, if both the effect size and accuracy are sufficiently large, we proceed to Step 3 for bottleneck table analysis, enabling a deeper investigation into the identified thresholds and critical factors influencing the outcome of interest. In Table 10, the effect sizes for green logistics and green finance are both categorized as having a "Medium Effect" size, with values of 0.29 and 0.24 respectively. This suggests that these variables moderately impact Green Innovation. In contrast, green technology exhibits a larger effect size of 0.45, classified as a "Large Effect." This indicates that Green Technology significantly influences Green Innovation within the studied context. However, the effect size for green work environment is notably smaller at 0.03, indicating a "Small Effect." This suggests that the influence of the green work environment on green innovation is relatively weak or negligible.

**Table 10:** Effect seizes (CE-FDH).

	Effect size (d)	P-value	Remarks
Green logistics	0.29	0.000	Medium effect
Green finance	0.24	0.000	Medium effect
Green technology	0.45	0.000	Large effect
Green work environment	0.03	0.653	Small effect

In the third step of our analysis, we utilize the bottleneck table to interpret the findings from multiple NCA. The bottleneck table, represented in Table 11, offers a structured overview of the required necessary levels of the conditions (green logistics, green finance, green technology, and green work environment) for different levels of the outcome (green innovation). The levels of the conditions and outcome are expressed as percentages of the observed range, ranging from 0 (minimum observed value) to 100 (maximum observed value), with 50 representing the midpoint^45^. According to the bottleneck table, when the outcome level (Y) is less than 10, there is no minimum requirement for any of the conditions (designated as NN, Not Necessary) for that outcome to occur. However, as the outcome level increases, minimum thresholds for each condition become necessary. For instance, when Y equals 10, a minimum level of 18.4 is required for green logistics, 3 for green finance, and 20.7 for green technology, while green work environment remains unnecessary. Similarly, when Y equals 90, higher minimum levels are required for green logistics (58.2), green finance (47.4), green technology (71.9), and green work environment (18.1). Notably, if any of these minimum levels is not attained in practice, the outcome Y = 100 will not occur, indicating that each condition can potentially act as a bottleneck for achieving the desired outcome.

**Table 11:** Bottleneck-values in percentage (NN = not necessary).

Y = green innovation	X = green logistics, green finance, green technology, green work environment			
	Green logistics	Green finance	Green technology	Green work environment
0	NN	NN	NN	NN
10	18.4	3.0	20.7	NN
20	18.4	6.8	23.0	NN
30	18.4	13.9	30.5	NN
40	18.4	13.9	32.6	NN
50	18.4	26.2	42.5	NN
60	31.2	26.2	52.2	NN
70	40.8	26.2	69.1	NN
80	40.8	34.0	71.9	NN
90	58.2	47.4	71.9	18.1
100	81.2	87.0	99.7	18.1

## Discussions and conclusions

The findings from PLS-SEM reveal a notable positive and significant impact of green finance, green logistics, and green technology on green innovation, while green work environment showed no significant effect. This observation could be rationalized through the lens of the RBV theory. According to RBV, a firm's competitive advantage and innovation potential stem from its unique resources and capabilities. Green finance facilitates the acquisition of financial resources necessary for investment in green projects, innovation, and technology adoption. Similarly, efficient green logistics practices optimize resource allocation and enhance operational efficiency, contributing to innovation by streamlining processes and reducing environmental impacts. Additionally, green technology investments enable firms to develop and implement innovative solutions that enhance sustainability and drive competitive advantage. Conversely, the non-significant impact of the green work environment may suggest that other factors, such as technology and financial resources, play a more decisive role in driving innovation outcomes within manufacturing firms. From a practical perspective, these findings underscore the importance of prioritizing investments in green finance, logistics, and technology to foster green innovation and sustainability within manufacturing firms.

Furthermore, the outcomes of PLS-SEM highlight a notable positive influence of green finance and green logistics on green technology, while the green work environment does not exhibit a significant effect. This observation is consistent with the Resource-Based View (RBV) theory, which suggests that a company's competitive advantage stems from its distinct resources and capabilities. Green finance empowers organizations to invest in the research, development, and implementation of green technologies, while effective green logistics practices optimize resource allocation and operational procedures, fostering the integration of green technologies across the supply chain. However, the absence of a significant impact from the green work environment implies that factors beyond organizational culture and workplace practices may hold greater sway in driving technological innovation. From a practical standpoint, these findings underscore the importance of prioritizing investments in green finance and logistics to propel technological innovation and sustainability efforts. Moreover, according to PLS-SEM, the mediating role of green technology in the relationships between green finance, green logistics, and green innovation signifies a crucial pathway through which investments in financial resources and efficient logistical practices translate into innovative outcomes. This suggests that companies can leverage their unique resources and capabilities to establish a competitive edge in the market.

The findings from fsQCA corroborate the results obtained from PLS-SEM, indicating that configurations involving green finance, green logistics, and green technology are sufficient conditions for high green innovation within manufacturing firms, irrespective of the green work environment. This consistency highlights the robustness of the observed relationships and underscores the importance of these factors in driving innovation outcomes. In both analyses, green finance and green logistics emerge as critical determinants of green innovation, emphasizing the significance of financial resources and efficient logistical practices in fostering sustainability-driven innovation. Additionally, the sufficiency of green technology further reinforces its pivotal role in facilitating innovation by enabling the adoption and integration of sustainable practices and technologies throughout the organization. However, the absence of green finance, green logistics, and green technology leads to low green innovation, underscoring the necessity of these factors for achieving meaningful progress towards sustainability goals. Together, these findings provide compelling evidence for the importance of prioritizing investments in green finance, logistics, and technology to drive innovation and sustainability within manufacturing firms, while also highlighting the need for a comprehensive approach to sustainability management that integrates multiple factors to maximize innovation potential.

The results of NCA provide valuable insights into the necessary conditions for green innovation within manufacturing firms, highlighting the significance of green logistics, green finance, and green technology while indicating that the green work environment is not a necessary factor. This suggests that while investments in financial resources, logistical efficiency, and technological advancement are crucial for driving innovation and sustainability, the organizational culture and workplace practices may not directly influence innovation outcomes. This has important implications for strategic decision-making and resource allocation within firms, as it underscores the need to prioritize investments in green finance, logistics, and technology to drive innovation, while potentially reallocating resources away from initiatives aimed solely at improving the work environment.

Moreover, the contrast between NCA and fsQCA demonstrates that NCA can pinpoint more essential conditions and delineate the necessary threshold for each condition to achieve a specific outcome, complementing the insights derived from fsQCA. This underscores the significance of employing diverse analytical methodologies to acquire a holistic comprehension of the factors influencing innovation outcomes and to guide strategic decision-making for sustainability initiatives. Through the integration of NCA alongside fsQCA, both researchers and practitioners can cultivate more nuanced understandings of the intricate dynamics of sustainability innovation. This, in turn, enables the development of targeted interventions and resource allocation strategies tailored to drive substantive advancements towards sustainability objectives within manufacturing enterprises.

Practical implications of the findings suggest that manufacturing firms should prioritize investments in green finance, green logistics, and green technology to drive innovation and sustainability initiatives. By allocating resources towards these key areas, firms can enhance their competitiveness, improve operational efficiency, and meet evolving environmental regulations and consumer preferences. Furthermore, the identification of the green work environment as a non-necessary factor implies that while fostering a supportive workplace culture is important for employee satisfaction and well-being, it may not directly influence innovation outcomes. Therefore, firms may need to reassess their resource allocation strategies and focus on initiatives that have a more direct impact on driving innovation and sustainability.

From a theoretical standpoint, the findings resonate with the RBV theory, which accentuates the significance of internal resources and capabilities in cultivating sustainable competitive advantages. Specifically, the results underscore the pivotal roles played by financial resources, logistical efficiency, and technological prowess in fostering innovation and sustainability within manufacturing enterprises. This bolsters the RBV assertion that firms possess the potential to harness their distinctive resources and capabilities to attain a competitive edge in the marketplace. Moreover, the recognition of green technology as a pivotal mediator in the interplay between green finance, green logistics, and green innovation further substantiates the RBV premise that valuable, rare, and difficult-to-replicate resources can confer sustainable competitive advantages. In sum, these findings enrich our comprehension of how firms can strategically manage their resources to propel innovation and sustainability, aligning closely with the foundational principles of RBV theory.

While this study provides valuable insights into the impact of green finance, green logistics, and green technology on green innovation, it is not without limitations. The research primarily focuses on manufacturing firms within a specific geographical context, which may limit the generalizability of the findings to other industries or regions. Additionally, the study does not fully explore the long-term effects of these green practices as the cross-sectional design of the study, leaving room for future research to examine their sustained impact on innovation outcomes over time. Future studies could also investigate the role of other factors, such as regulatory environments or consumer demand, in shaping the relationship between green practices and innovation, providing a more comprehensive understanding of how to drive sustainable business practices across various contexts.

This study offers valuable insights into the drivers of green innovation within manufacturing firms. Our findings indicate that green logistics and green finance exert a positive and significant influence on green innovation, while the impact of the green work environment, though positive, was not statistically significant. Moreover, green technology was identified as a significant mediator in the relationships between green finance, green logistics, and green innovation. Additionally, the application of fsQCA revealed the sufficiency of green finance, green logistics, and green technology in fostering high levels of green innovation, irrespective of the green work environment's level. These results underscore the importance of investing in green finance, logistics, and technology to propel sustainability-oriented innovation within manufacturing firms.

This study makes four significant contributions to the literature on green innovation within manufacturing firms. Firstly, by developing a comprehensive model of green innovation, the study provides a framework for understanding the key drivers and mechanisms underlying sustainability-driven innovation. This model offers valuable insights into the interrelationships between green logistics, green finance, green work environment, green technology, and green innovation, facilitating a holistic approach to sustainability management within manufacturing firms. Sufficient conditions with configurations are discovered for high green innovation that led to a comprehensive green innovation model. Secondly, the identification of green technology as a key mediator in the relationships between financial, logistical, and environmental factors and innovation outcomes adds to our understanding of the mechanisms driving sustainability-driven innovation. This highlights the pivotal role of technological advancement in translating investments in green finance and logistics into tangible innovation outcomes.

Thirdly, the study contributes theoretically by integrating green technology into the RBV theory, highlighting its importance as a strategic resource for gaining competitive advantage in sustainability-driven markets. This underscores the relevance of RBV theory in explaining the dynamics of innovation and sustainability within manufacturing firms. Lastly, the application of a multi-method approach, including PLS-SEM, fsQCA, and NCA enhances the robustness and comprehensiveness of the findings. By integrating quantitative and qualitative methods, the study provides a nuanced understanding of the complex relationships between various factors influencing green innovation, offering valuable insights for both researchers and practitioners in the field of sustainability management.

## Supplementary Information

Supplementary Tables.

## Data Availability

Data will be made available on reasonable request through corresponding author.
