# Author Correction: Exploring the synergy of logistics, finance, and technology on innovation

**DOI:** 10.1038/s41598-024-76146-x

**Published:** 2024-10-18

**Authors:** Chunfang Wang, Md. Mominur Rahman, Abu Bakkar Siddik, Zheng Guang Wen, Farid Ahammad Sobhani

**Affiliations:** 1 College of E-Commerce and Logistics, Henan Polytechnic, Zhengdong New District, Zhengzhou, 450018 Henan Province China; 2 Bangladesh Institute of Governance and Management (BIGM), University of Dhaka (Affiliated), Dhaka, 1207 Bangladesh; 3 School of Management, University of Science and Technology of China, Jinzhai Road, Hefei, 230026 China; 4 School of Economics and Management, Shaanxi University of Science and Technology, Xi’an, 710021 Shaanxi China; 5 School of Business and Economics, United International University, Dhaka, 1212 Bangladesh

Correction to: *Scientific Reports*10.1038/s41598-024-72409-9, published online 19 September 2024

The original version of this Article contained an error in the Funding section.

“This study was partially funded by the Institute for Advanced Research Publication Grant of United International University, Ref. No.: IAR-2024-Pub-050.”

now reads:

“This study was partially funded by the Institute for Advanced Research Publication Grant of United International University, Ref. No.: IAR-2024-Pub-057"

The original Article has been corrected.

