# Supplementary material for: Exploring the synergy of logistics, finance, and technology on innovation
Source: Sci Rep. 2024 Sep 19;14:21918. doi: 10.1038/s41598-024-72409-9 (PMC11413022; doi:10.1038/s41598-024-72409-9)
Supplement: Supplementary file 1 — Supplementary Tables. [file 41598_2024_72409_MOESM1_ESM.docx]

# Appendix

Table A1. Variables and their sources

| **Constructs** | **Items** | **Sources** |
| --- | --- | --- |
| **1. Green Logistics** |  |  |
| ***1.1. Eco-friendly Transportation Adoption*** | Our company actively seeks out eco-friendly transportation options for shipping and logistics. | 10,13,28 |
|  | We prioritize the use of low-emission vehicles for transportation purposes. |  |
|  | Our company invests in technologies that reduce the environmental impact of transportation. |  |
| ***1.2. Sustainable Packaging Implementation*** | We utilize recyclable materials for our product packaging whenever possible. |  |
|  | Our company has implemented measures to reduce packaging waste. |  |
|  | We strive to use biodegradable packaging materials to minimize environmental harm. |  |
| ***1.3. Reverse Logistics Integration*** | Our company has established processes for the efficient return and recycling of products. |  |
|  | We actively engage in the reuse and refurbishment of returned products. |  |
|  | Our reverse logistics system is designed to minimize waste and maximize resource efficiency. |  |
| ***1.4. Supply Chain Carbon Footprint Efficiency*** | We monitor and track the carbon footprint of our entire supply chain. |  |
|  | Our company implements measures to reduce greenhouse gas emissions throughout the supply chain. |  |
|  | We collaborate with suppliers to adopt environmentally friendly practices and reduce carbon emissions. |  |
| **2. Green Finance** |  |  |
| ***2.1. Green Investment Fund Availability*** | Our company allocates financial resources specifically for green initiatives and sustainability projects. | 21,30,31 |
|  | We have dedicated funds for investing in environmentally friendly technologies and practices. |  |
|  | Our company actively seeks out investment opportunities that align with our environmental goals. |  |
| ***2.2. Corporate Green Financing Policies*** | Our company has established policies to encourage green financing and sustainable investment. |  |
|  | We offer incentives and financial support for projects that promote environmental sustainability. |  |
|  | We integrate environmental criteria into our financial decision-making processes. |  |
| ***2.3. Eco-friendly Funding Accessibility*** | Our company provides accessible funding options for eco-friendly projects and initiatives. |  |
|  | We offer financial assistance to employees or partners who implement green practices. |  |
|  | We prioritize funding allocations towards projects that have a positive environmental impact. |  |
| ***2.4. Environmental Incentives*** | Our company rewards employees for implementing environmentally friendly practices. |  |
|  | We offer incentives for suppliers who adopt sustainable and eco-friendly measures. |  |
|  | We provide incentives for customers who choose environmentally friendly products or services. |  |
| **3. Green Work Environment** |  |  |
| ***3.1. Eco-friendly Workplace Practices Adoption*** | Our company promotes recycling and waste reduction in the workplace. | 15,16 |
|  | We encourage energy-saving practices such as turning off lights and equipment when not in use. |  |
|  | Our workplace actively promotes the use of reusable materials and products. |  |
| ***3.2. Green Building Standards Implementation*** | Our company adheres to green building standards in the design and construction of our facilities. |  |
|  | We implement energy-efficient systems and technologies in our buildings. |  |
|  | Our company prioritizes the use of sustainable materials and practices in building construction and maintenance. |  |
| ***3.3. Telecommuting Promotion*** | Our company supports telecommuting options to reduce employee commuting and carbon emissions. |  |
|  | We provide resources and infrastructure to facilitate remote work arrangements. |  |
|  | We actively promote telecommuting as part of our sustainability initiatives. |  |
| ***3.4. Employee Sustainability Engagement*** | Our employees are actively engaged in sustainability initiatives and activities. |  |
|  | We encourage employee participation in environmental awareness campaigns and events. |  |
|  | Our company fosters a culture of sustainability, where employees are encouraged to contribute ideas and initiatives for environmental improvement. |  |
| **4. Green Technology** | Our company invests in cutting-edge technologies that reduce environmental impact. | 29,34,35 |
|  | We prioritize the adoption of renewable energy technologies in our operations. |  |
|  | We integrate innovative technologies to optimize resource efficiency and minimize waste generation. |  |
|  | Our company actively researches and develops green technologies to address environmental challenges. |  |
| **5. Green Innovation** | Our company continuously seeks innovative solutions to reduce our environmental footprint. | 6,33,34,39 |
|  | We encourage employees to propose and implement green innovations within our operations. |  |
|  | Our company fosters a culture of creativity and experimentation to drive green innovation. |  |
|  | We collaborate with partners and stakeholders to develop and implement new environmentally sustainable practices and products. |  |

Table A2. Truth table minimization

| GRF | GRL | GRT | GWE | number | GRIN | raw consist. | PRI consist. | SYM consist |
| --- | --- | --- | --- | --- | --- | --- | --- | --- |
| 1 | 1 | 1 | 0 | 92 | 1 | 0.99149 | 0.973453 | 0.977129 |
| 1 | 1 | 1 | 1 | 71 | 1 | 0.988675 | 0.96384 | 0.968216 |
| 1 | 0 | 1 | 0 | 12 | 1 | 0.986867 | 0.909358 | 0.909359 |
| 1 | 0 | 1 | 1 | 9 | 1 | 0.985533 | 0.896656 | 0.901678 |
| 0 | 1 | 1 | 0 | 5 | 1 | 0.980701 | 0.814581 | 0.82198 |
| 0 | 0 | 1 | 0 | 6 | 1 | 0.975868 | 0.728144 | 0.728145 |
| 1 | 1 | 0 | 0 | 5 | 1 | 0.971803 | 0.609657 | 0.609659 |
| 0 | 1 | 1 | 1 | 28 | 1 | 0.971354 | 0.816762 | 0.837694 |
| 0 | 0 | 1 | 1 | 6 | 1 | 0.968088 | 0.704122 | 0.709284 |
| 1 | 1 | 0 | 1 | 5 | 1 | 0.962453 | 0.476881 | 0.476881 |
| 1 | 0 | 0 | 1 | 4 | 1 | 0.951756 | 0.387288 | 0.389155 |
| 0 | 1 | 0 | 0 | 3 | 1 | 0.941344 | 0.223239 | 0.223239 |
| 1 | 0 | 0 | 0 | 17 | 1 | 0.936643 | 0.388501 | 0.395389 |
| 0 | 1 | 0 | 1 | 12 | 1 | 0.921045 | 0.173873 | 0.174037 |
| 0 | 0 | 0 | 0 | 69 | 0 | 0.780903 | 0.062857 | 0.063649 |
| 0 | 0 | 0 | 1 | 85 | 0 | 0.768373 | 0.054567 | 0.054749 |
